# Genetic Association Between TMPRSS2 Polymorphisms and COVID-19 Severity in Brazilian Health Workers

**DOI:** 10.3390/v17121587

**Published:** 2025-12-05

**Authors:** Alysson Fellipe Costa Telles, Bearli Souza Menezes Junior, Cliomar Alves dos Santos, Maria Rosa Melo Alves, Ludmila Oliveira Carvalho Sena, Rosana Cipolotti

**Affiliations:** 1 Programa de Pós-graduação em Ciências da Saúde da Universidade Federal de Sergipe, Aracaju 49107-230, Brazil; rosa.alves.biomed@gmail.com (M.R.M.A.); ludmilacarvalho25@gmail.com (L.O.C.S.); rosnaci@yahoo.com (R.C.); 2 Fundação de Saúde Parreiras Horta—Lacen SE, Aracaju 49020-590, Brazil; bearlijr.biomed@gmail.com (B.S.M.J.); cliomarsantos@gmail.com (C.A.d.S.)

**Keywords:** single-nucleotide polymorphism, SARS-CoV-2, occupational health, genetic susceptibility, enzymological regulation of gene expression

## Abstract

The diversity of clinical presentations and outcomes of COVID-19 suggests the influence of host-intrinsic factors that modulate the infectious process. Therefore, a study was conducted with professionals from a hospital in the state of Sergipe, in the Northeast region of Brazil, aiming to identify in this population the effect of rs12329760 and rs2070788, SNPs of the TMPRSS2 enzyme that facilitates the infectious process. Recruitment of the 363 participants followed a non-probabilistic method using a QR code that led to the Informed Consent Form (ICF) and a clinical–epidemiological questionnaire based on self-reported information on the number of positive tests, the presence/absence of symptoms, and severity. Buccal epithelial cells were collected, DNA was extracted using a silica column, and SNP amplification was performed by qPCR. The data were processed using PSPP software, using chi-squared tests for associations in three statistical genetic models (additive, dominant, and recessive). The results showed that, in this population, rs12329760 did not influence any of the outcomes, while rs2070788 was significant in both the additive and recessive models. The action of the G allele is evident in the most severe cases, and it is associated with increased TMPRSS2 expression and potentially increased viral entry efficiency.

## 1. Introduction

The diverse clinical presentation of COVID-19 following the same host exposure, as well as the occurrence of low-probability outcomes, such as young individuals without comorbidities progressing to death, and elderly individuals with asymptomatic infections, raises important questions, such as the possible influence of genetic factors that are intrinsic to everyone [1,2,3]. In this context, the presence of Single-Nucleotide Polymorphisms (SNPs) in genes encoding important molecules in the infection process, such as ACE2 and TMPRSS2, may affect these molecules structurally and functionally, altering their expression profiles both quantitatively and qualitatively. ACE2 acts as a direct receptor for SARS-CoV-2, while TMPRSS2 acts as a catalyst for viral fusion with the host membrane, with an increase in functionally active and contributes effectively to viral entry [4,5,6,7].

The gene encoding TMPRSS2 is located on chromosome 21 at 21q22.3. It is highly polymorphic, considering the variations in frequencies according to the studied population, and the literature presents two SNPs as potential modulators of the infectious process caused by SARS-CoV-2: rs2070788 and rs12329760 [2,8,9,10].

rs2070788 is in intron 11-12, characterized by the substitution of a Guanine for an Adenine at position 587, a region with no known regulatory function to date, but it appears to be related to increased TMPRSS2 expression when the G allele is present; this relationship is probably attributed to linkage disequilibrium with another SNP located in the regulatory region. rs12329760 is characterized by a substitution of the T allele for C at position 589 of the gene, a promoter region, and is therefore classified as a missense mutation, replacing a valine with a methionine at position 160, the T allele appears to be related to lower expression of the TMPRSS2 protein, with moderate catalytic activity [2,8,9,11,12].

Despite all the knowledge produced about SNPs, the different ethnicities worldwide and the diverse ancestry profiles demonstrate the importance of understanding the genetic profile linked to people inhabiting different continents, and how genotypes affect the phenotypic characteristics manifested by each ethnic group [5,9,13,14]. Brazil is a country of mixed race, with European, African, and indigenous influences, and due to its large territorial extension, ancestry frequencies vary according to the region of the country [15,16].

This study aimed to relate the presence of the polymorphisms rs2070788 and rs12329760 in a group of health workers from Sergipe (the smallest state in Brazil, located in the Northeast region) with susceptibility or resistance to SARS-CoV-2 infection, and with the clinical severity of the infectious event. Thus, this study aims to contribute to the scientific knowledge about the effects of these SNPs worldwide and to propose therapeutic targets and genetic markers of vulnerability for the risk stratification of patients infected by SARS-CoV-2. This is the first study to address the genetic association of these SNPs in this population, which is characterized by intense miscegenation and, consequently, great phenotypic diversity.

## 2. Materials and Methods

The study involved participants over 18 years of age, linked to the University Hospital of Sergipe, recruited using a non-probabilistic convenience sampling method, as the research group is part of this institution and has easy access to professionals from various hospitals and administrative activities. The hospital, which currently has 3031 employees registered in its own institutional system, acted as a reference in the State of Sergipe regarding assistance to people infected with SARS-CoV-2 during the pandemic period between 2020 and 2021.

Recruitment occurred by sending a QR Code in virtual and physical formats, directing participants to the Informed Consent Form (ICF) and Clinical and Epidemiological Questionnaire (CEQ). The latter consisted of objective and subjective questions about the symptom profile presented by patients when they were clinically suspected of COVID-19 or underwent molecular investigation for SARS-CoV-2 by qPCR. Furthermore, participants were asked about the number of positive tests they had, as well as their demographic and social data (sex, age, city of residence, and race).

All clinical and laboratory data were obtained by self-report, following the proposal of Tug et al., 2023 [15], as severity stratification follows strict clinical criteria, and this study did not have access to the participants’ medical records. The severity classification considered the reported symptoms and the need to seek medical attention. Likewise, the number of positive tests could only be confirmed by checking reports in a service that was decentralized throughout Brazilian territory [16,17,18,19].

Biological material collection was performed using swabs in the oral mucosa region, following the protocol of the study conducted by Araujo et al. in 2021 [20], which showed that the quality of the DNA extracted remains very good for molecular analyses using these cells. The samples were sent at room temperature to the molecular biology laboratory of the Public Health Laboratory of the State of Sergipe (Lacen-SE) on the same day, where they were vortexed, followed by discarding the swab. tubes containing the cells were stored at 2–8 °C until the start of the genetic study [21,22].

**Figure 1:**
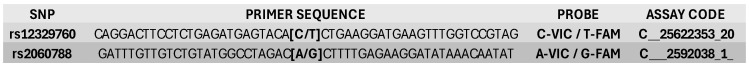
Characterization of the primer and probe sequences related to the molecular analysis by qPCR for the SNPs rs12329760 and rs2070788 of the TMPRSS2 enzyme. Source: https://www.thermofisher.com/order/genome-database/details/genotyping/C__25622353_20 and https://www.thermofisher.com/order/genome-database/details/genotyping/C___2592038_1_ (accessed on 6 November 2025).

DNA extraction methodology using a silica column from Qiagen^®^ (Venlo, The Netherlands) was performed according to the manufacturer’s guidelines. For qPCR, the master mix used was from Promega^®^ (Madison, WI, USA), and the primers were acquired from Thermo Scientific^®^ (Waltham, MA 02451, USA), which designed the primer sequences and labeled them with the respective probes. Figure 1 represents the nucleotide sequence of each of the targets, as well as the representation of the polymorphisms (C/T for rs12329760 and A/G for rs2070788), with the probe marking each allele.

The cycling program in the QuantStudio 5 equipment from Thermo Scientific^®^ included a pre-amplification phase (Hold Stage), with incubation at 60 °C for 1 min for enzymatic activation, followed by 95 °C for 10 min for the first denaturation, ensuring the presentation of single-stranded DNA for the reaction. The amplification phase consisted of 40 cycles at 95 °C for 15 s and 60 °C for 1 min. Then, the post-read phase finished the run, with the reaction at 60 °C for one final minute.

The results were expressed in an allelic discrimination plot and evaluated using three statistical genetic models. The additive model assumes a linear increase based on the number of copies of the related allele present in the genotyping,; the dominant model assumes the hypothesis that, regardless of one or two copies of the allele, the risk increases, and the recessive model presents the hypothesis that two copies of the allele are necessary to alter the risk related to the characteristic/disease.

All data were categorized and analyzed using the PSPP software—“Portable Statistical Processing Program” version 2.0, where they were described in absolute and relative frequencies, in addition to association analyses using Fisher’s exact test for quadratic tables and Pearson’s chi-square for non-quadratic tables. The standardized confidence interval was 95% to show statistical significance (*p* < 0.05), and the sample calculation proposed 342 participants to infer whether the results were linked to chance or had some relation between their frequencies. The entire theoretical framework and discussion of the results of this research were based on articles produced in the last 5 years, except one from 2015, which is important for the discussion.

## 3. Results

A total of 363 staff members from the University Hospital of Sergipe accessed the QR code, completed the clinical-epidemiological questionnaire (CEQ), and provided oral mucosa cell samples. The group predominantly comprised females (76%) of working age (30–59 years), with 58.7% self-identifying as mixed race (Pardo). The vast majority (83.5%) resided in Aracaju, the state capital, where the University Hospital is located, and the others lived in surrounding cities.

Regarding their role in the hospital, there was representative participation from three categories: administrative workers (26.4%), indirect care staff (43.3%, comprising medical, nursing, and dental professionals), and direct care staff (30.3%, comprising professionals from other care areas). The data are presented in Table 1.

Genotyping for the SNPs rs2070788 and rs12329760 was evaluated by representing the Minor Allele Frequency (MAF), compared with information from the 1000 Genomes Project for both the Americans and Latin American populations (NCBI, n.d., 2024) using the chi-square test. The C allele was the most prevalent for rs12329760 and the A allele for rs2070788 (NCBI, n.d., 2024) at frequencies that did not present differences between the studied population and the reference data available at NCBI, representing that the studied group is in Hardy–Weinberg Equilibrium (HWE), as described in Table 2.

The results showed no association between the two SNPs and the number of positive tests, suggesting that neither rs12329760 nor rs2070788 influences susceptibility to SARS-CoV-2 in any of the three genetic models for this population, as described in Table 3.

Of the 363 participants, 43 reported being asymptomatic 160 reported mild events treated at home, and 160 reported moderate symptoms requiring referral to healthcare services; there were no reports of a need for intensive care. Regarding the “severity” variable, the genetic data suggest that rs12329760 does not influence the severity of symptoms presented by the participants in this study. However, the presence of the G allele of the rs2070788 SNP was associated with more severe events in both the additive (*p* = 0.016) and recessive (*p* = 0.004) models. Thus, this study highlights the importance of this polymorphism in this population in terms of its frequency and influence on modulating symptoms, infectious conditions, and clinical progression. Data on the association between genotyping and severity are presented in Table 4.

In a subsequent analysis of severity with only symptomatic individuals, a statistical association was proposed between the groups with mild and moderate symptoms. A narrowing of this relationship was observed, with a *p*-value of 0.004 for the additive model and 0.001 for the recessive model, demonstrating the strength of this association in a more uniform group, as shown in Table 5.

When assessing the genetic significance of the G allele of rs2070788 in the additive model, which presents a combined effect, with the homozygous condition being associated with twice the allele’s effect on this trait, the Bonferroni-corrected chi-square test demonstrated the relationship GG > AG > AA (*p*-value = 0.001; 0.004; and 0.442, respectively), reinforcing the effect of the G allele on the severity condition. These data are supported by a similar study, which found the same relationship [23].

Considering the dichotomization of severity, with the exclusion of asymptomatic individuals, a binary logistic regression model was proposed to support the results found for the recessive model and demonstrated a *p*-value of 0.001 and an OR of 2.22 for the presentation of more severe symptoms related to the presence of the G allele.

## 4. Discussion

The data on sex and age were consistent with the profile of hospital workers in Brazil, even though recruitment was performed using a non-probabilistic method. Despite the growing elderly population in the labor market, the data remain conservative regarding age [21,22,24,25]. The race profile also follows the parameters of the latest census conducted by the Brazilian Institute of Geography and Statistics [26], showing intense miscegenation resulting from the influence of the country’s historical colonization [27]. The state capital hosts most participants, followed by cities bordering Aracaju, given that Sergipe is the smallest state in the federation, which allows for the flow of people within a short time frame [28]. The adherence of the three occupational spheres demonstrates the effectiveness of QR Code recruitment methods, reflected by the excellent reach and participation.

Regarding rs12329760, the scientific community has presented heterogeneous results. Two studies conducted in Egypt are discordant when assessing the effects of the T allele as a predictor of severity, where one portrays the symptom-enhancing effect of this allele, while the other claims a severity-reducing effect, in the same population, thus dealing with people of the same ancestry profile [17,28].

A Spanish study, a known ancestor of the Sergipan population, reproduced the results of the present study, showing no statistical impact on the studied outcome variables [10]. In contrast, a study conducted in the UK involving symptomatic and severely ill patients highlighted the protective factor of the T allele, characterizing the moderate catalytic activity of the TMPRSS2 enzyme, mainly in the recessive model. Of the 137 variants evaluated as harmful to the structure and/or function of TMPRSS2, 136 were rare, making their association with COVID-19 severity unlikely. Only rs12329760 proved efficient owing to functional changes in the protein structure, dismissing any association with an eQTL—Expression Quantitative Trait Loci available on GTEx [29].

However, the same study drew attention to the fact that TMPRSS2, since the COVID-19 pandemic began, has been studied as an activator of the fusion between SARS-CoV-2 and host cells. However, it is necessary to examine this protein broadly and better understand its pathophysiology, as it is a soluble protease that can act on other substrates and may be more directly related to inflammatory processes mediated by the production of prostaglandins and cytokines in the lungs and other organs. The findings regarding the Bonferroni test for the additive model are supported by a similar study, which found the same relationship [29].

The results for rs2070788 are more homogeneous regarding its role as an important marker of severity and susceptibility to SARS-CoV-2, according to the ancestry profile of the studied populations. A publication of Native Americans highlighted the distribution profile of these alleles, with rs2070788 being strongly associated with ancestry in this population [11], as well as research on fatal cases in India [30], which cites Americans as having a higher frequency of rs2070788 than Africans and Asians, which could explain the higher incidence of deaths in Americans than in the other cited populations.

A study conducted with 402 patients in Petrolina (PE, Brazil) found similar results, with rs12329760 showing no difference between groups and rs2070788 showing the G allele as a severity factor, even associated with death, owing to the higher expression of TMPRSS2, which is promoted in lung tissue. Consequently, there is an increase in the cleavage of ACE2 and SARS-CoV-2, this event being decisive for infectivity and, consequently, patient outcomes. The absence of a correlation with rs12329760 is explained in this work, resulting in a structural change in the protein that does not necessarily affect its expression levels [8].

However, Kehdy et al., 2021 [11] reinforced the need to evaluate Linkage Disequilibrium (LD) with other alleles in the same gene, which might also intervene in the pathophysiology of COVID-19, citing rs383510, whose relationship with the SNPs is also evidenced by Cheng et al., 2015 [23], who defended the hypothesis that rs2070788, although located in an intronic region, does not represent a regulatory region and does not directly affect TMPRSS2 expression. rs383510, in contrast, is in a putative regulatory region, shows high linkage disequilibrium with rs2070788 in Asian populations, and appears in genetic studies as a cis-eQTL for lung tissue [11,23].

Another scientific publication from Germany reinforces the information about the predisposition to severe infections by the G allele, stating that the scientific basis of this action is not well clarified, since rs2070788 is in a non-regulatory intron region and promotes increased enzyme expression. However, this study found no statistical correlation with either SNP (rs12329760 and rs2070788), but emphasized the difference in MAF depending on ethnicity, meaning that the variants were not relevant for a study with Germans, but cannot be definitively dismissed and should be associated with the participants’ ancestry profile. Considering the small German influence on the colonization of Sergipe, the difference in results makes sense [9].

The clinical implications of these findings deserve consideration. The identification of rs2070788 as a possible marker of disease severity could potentially guide risk-stratification protocols in healthcare settings, particularly considering the occupational exposure risks faced by healthcare professionals. However, despite the results found, the implementation of genetic screening would require careful analysis of cost-effectiveness, ethical implications, and the need for larger validation studies in diverse populations, given the recruitment strategy of this study (which may bias the results) and the mixed-race profile of the study population, which may be affected by the additional influence of other polymorphisms involved.

## 5. Conclusions

Based on the results, it can be concluded that the clinical manifestation profile of COVID-19 is multifactorial and varied, and may be influenced by sociodemographic variables such as sex, age, and pre-existing comorbidities, as well as by genetic components involved in the adhesion and fusion of enzymes with human cells, such as TMPRSS2. Although rs12329760 did not show differences between the groups, rs2070788 appeared in this population as a factor associated with more severe symptomatic cases; however, the mechanisms by which this occurs require further clarification. After larger cohort or Case–Control studies that better demonstrate this relationship, it may be used as a future therapeutic target, as well as for identifying more susceptible individuals, thereby acting as a risk stratification tool. It is important to emphasize that genetic assessments should not be generalized to any population; their ancestry profile should always be considered for better associations.

## Figures and Tables

**Table 1:** Sociodemographic and professional characteristics of study participants (n = 363).

Variable	Categories	Abs	%
Sex	Male	87	24
	Female	276	76
Age	Young adults (≤29 years)	30	8.3
	Adults (30–59 years)	325	89.5
	Elderly (≥60 years)	8	2.2
Race/Color	Black	49	13.5
	Mixed race (Pardo)	213	58.7
	White	94	25.9
	Indigenous	1	0.3
	Asian (yellow)	5	1.4
	No response	1	0.3
City of residence	Aracaju (capital)	303	83.5
	Nossa Senhora do Socorro	16	4.4
	Barra dos Coqueiros	23	6.3
	São Cristóvão	10	2.8
	Other cities of Sergipe	11	3
Role in the hospital	Administrative/Management	96	26.4
	Indirect care	157	43.3
	Direct care	110	30.3

Table from author.

**Table 2:** Distribution of allele frequencies of the sample from this study and from reference populations, based on the 1000 Genomes Project.

SNP	Alleles	Allele Frequency	Allelic Frequency in the Americas	Allelic Frequency in Latin America	*p* Value
rs12329760	C	0.81	0.85	0.84	0.199
	T	0.19	0.15	0.16	
rs2070788	A	0.57	0.51	0.56	0.223
	G	0.43	0.49	0.44	

Table from author.

**Table 3:** Association between genotyping in the 3 statistical genetic models and number of positive tests.

SNP	Associated Allele	Genetic Model	Genotyping	No Positive Tests	Between 1 and 2 Positive Tests (Average)	3 or More Positive Tests	*p* Value
rs12329760	C	Additive	CC	46 (12.7%	128 (35.3%)	68 (18.7%)	0.839
			CT	16 (4.4%)	62 (17.1%)	26 (7.2%)	
			TT	3 (0.8%)	9 (2.5%)	5 (1.4%)	
		Dominant	TT	3 (0.8%)	9 (2.5%)	5 (1.4%)	0.979
			CC + CT	62 (17.1%)	190 (52.3%)	94 (25.9%)	
		Recessive	TT + CT	19 (5.2%)	71 (19.6%)	31 (8.5%)	0.558
			CC	46 (12.7%)	128 (35.3%)	68 (18.7%)	
rs2070788	A	Additive	AA	18 (5%)	65 (17.9%)	34 (9.4%)	0.855
			AG	36 (9.9%)	97 (26.7%)	46 (12.7%)	
			GG	11 (3%)	37 (10.2%)	19 (5.2%)	
		Dominant	GG	11 (3.0)	37 (10.2%)	19 (5.2%)	0.933
			AG + AA	54 (14.9%)	162 (44.6%)	80 (22.0%)	
		Recessive	AG + GG	47 (12.9%)	134 (36.9%)	65 (17.9%)	0.66
			AA	18 (5.0%)	65 (17.9%)	34 (9.7%)	

Table from author.

**Table 4:** Association between genotyping in the 3 statistical genetic models and symptom severity, using a 95% confidence interval.

SNP	Associated Allele	Genetic Model	Genotyping	No Symptoms n = 43	Mild Symptoms n = 160	Moderate Symptoms n = 160	*p* Value
rs12329760	C	Additive	CC	32 (8.8%)	104 (28.7%)	106 (29.2%)	0.818
			CT	9 (2.5%)	48 (13.2%)	47 (12.9%)	
			TT	2 (0.6%)	8 (2.2%)	7 (1.9%)	
		Dominant	TT	2 (0.6%)	8 (2.2%)	7 (1.9%)	0.966
			CC + CT	41 (11.3%)	152 (41.9%)	153 (42.1%)	
		Recessive	TT + CT	11 (3%)	56 (15.4%)	54 (14.9%)	0.503
			CC	32 (8.8%)	104 (28.7%)	106 (29.2%)	
rs2070788	A	Additive	AA	16 (4.4%)	37 (10.2%)	64 (17.6%)	**0.016**
			AG	18 (5%)	93 (25.6%)	68 (18.7%)	
			GG	9 (2.5%)	30 (8.3%)	28 (7.7%)	
		Dominant	GG	9 (2.5%)	30 (8.3%)	28 (7.7%)	0.869
			AG + AA	34 (9.4%)	130 (35.8%)	132 (36.4%)	
		Recessive	AG + GG	27 (7.4%)	123 (33.9%)	96 (26.4%)	**0.004**
			AA	16 (4.4%)	37 (10.2%)	64 (17.6%)	

Table from author.

**Table 5:** Association between genotyping in the 3 statistical genetic models and symptom severity in symptomatic cases, using a 95% confidence interval.

SNP	Associated Allele	Genetic Model	Genotyping	Mild Symptoms n = 160	Moderate Symptoms n = 160	*p* Value
rs12329760	C	Additive	CC	104 (32.4%)	107 (33.3%)	0.953
			CT	48 (15%)	47 (14.6%)	
			TT	8 (2.5%)	7 (2.2%)	
		Dominant	TT	8 (2.4%)	7 (2.2%)	0.791
			CC + CT	152 (47.4%)	154 (48%)	
		Recessive	TT + CT	56 (17.4%)	54 (16.8%)	0.814
			CC	104 (32.4%)	107 (33.4%)	
rs2070788	A	Additive	AA	37 (11.5%)	64 (19.9%)	**0.004**
			AG	93 (29.1%)	68 (21.2%)	
			GG	30 (9.3%)	29 (9%)	
		Dominant	GG	30 (9.3%)	29 (9%)	0.772
			AG + AA	130 (40.5%)	132 (41.2%)	
		Recessive	AG + GG	123 (38.3%)	97 (30.2%)	**0.001**
			AA	37 (11.5%)	64 (2.0%)	

Table from author.

## Data Availability

For ethical reasons, the data related to this study cannot be made publicly available, given that the Informed Consent Form does not allow for such sharing beyond the research group, and provides guarantees regarding the anonymity of the participant and the confidentiality of the information provided. However, if absolutely necessary for the validation of the results, they may be requested from the corresponding author, in compliance with the confidentiality terms and Brazilian data protection legislation (Law No. 13,709/2018—LGPD).

## Abbreviations

The following abbreviations are used in this manuscript:

SNP	Single-Nucleotide Polymorphism
TMPRSS2	Transmembrane protease serine 2
ACE2	Angiotensin-converting enzyme
